# Points to consider in cardiovascular disease risk management among patients with rheumatoid arthritis living in South Africa, an unequal middle income country

**DOI:** 10.1186/s41927-020-00139-2

**Published:** 2020-06-16

**Authors:** Ahmed Solomon, Anne E. Stanwix, Santos Castañeda, Javier Llorca, Carlos Gonzalez-Juanatey, Bridget Hodkinson, Benitha Romela, Mahmood M. T. M. Ally, Ajesh B. Maharaj, Elsa M. Van Duuren, Joyce J. Ziki, Mpoti Seboka, Makgotso Mohapi, Barend J. Jansen Van Rensburg, Gareth S. Tarr, Kavita Makan, Charlene Balton, Aphrodite Gogakis, Miguel A. González-Gay, Patrick H. Dessein

**Affiliations:** 1 Rheumatology Department, Charlotte Maxeke Johannesburg Academic Hospital, Faculty of Health Sciences, University of Witwatersrand, 80 Scholtz Road, Norwood, Johannesburg, 2190 South Africa; 2 Rheumatology Department, Hospital de la Princesa, IIS-Princesa, Cátedra UAM-ROCHE, EPID-Future, Department of Medicine, Universidad Autónoma de Madrid (UAM), Madrid, Spain; 3 Universidad de Cantabria – IDIVAL, CIBER Epidemiologia y Salud Pública (CIBERESP), Santander, Spain; 4 Cardiology Department, University Hospital Lucus Augusti, Lugo, Spain; 5 Rheumatology Department, University of Cape Town and Groote Schuur Hospital, Cape Town, South Africa; 6 Rheumatology Unit, Wilgeheuwel Hospital, Johannesburg, South Africa; 7 Rheumatology Department, Steve Biko Academic Hospital, University of Pretoria, Pretoria, South Africa; 8 Rheumatology Unit, Westville Hospital and University of KwaZulu-Natal, Durban, South Africa; 9 Rheumatology Division, Department of Medicine, Sefako Makgatho Health Sciences University, Pretoria, South Africa; 10 Rheumatology Department, Universitas Hospital, Bloemfontein, Free State South Africa; 11 Rheumatology Department, Tygerberg Hospital, Faculty of Health Sciences, Physiological Sciences Department, Stellenbosch University, Stellenbosch, Western Cape South Africa; 12 Rheumatology Department, Chris Hani Baragwanath Hospital, Johannesburg, South Africa; 13 Radiology Unit, Rivonia Road Medical Centre, Morningside, Johannesburg, South Africa; 14 Cardiovascular Pathophysiology and Genomics Research Unit, School of Physiology, Faculty of Health Sciences, University of Witwatersrand, Johannesburg, South Africa; 15 Division of Rheumatology and Epidemiology, Genetics and Atherosclerosis Research Group on Systemic Inflammatory Diseases, Hospital Universitario Marqués de Valdecilla, Instituto de Investigación Marqués de Valdecilla (IDIVAL), Spain; University of Cantabria, Santander, Spain; 16 School of Physiology and School of Clinical Medicine, Faculty Health Sciences, University of Witwatersrand, Johannesburg, South Africa; 17 Free University and University Hospital, Brussels, Belgium

**Keywords:** Cardiovascular disease risk management, Rheumatoid arthritis, Low to middle income countries, South Africa

## Abstract

**Background:**

It is plausible that optimal cardiovascular disease (CVD) risk management differs in patients with rheumatoid arthritis (RA) from low or middle income compared to high income populations. This study aimed at producing evidence-based points to consider for CVD prevention in South African RA patients.

**Methods:**

Five rheumatologists, one cardiologist and one epidemiologist with experience in CVD risk management in RA patients, as well as two patient representatives, two health professionals and one radiologist, one rheumatology fellow and 11 rheumatologists that treat RA patients regularly contributed. Systematic literature searches were performed and the level of evidence was determined according to standard guidelines.

**Results:**

Eighteen points to consider were formulated. These were grouped into 6 categories that comprised overall CVD risk assessment and management (*n* = 4), and specific interventions aimed at reducing CVD risk including RA control with disease modifying anti-rheumatic drugs, glucocorticoids and non-steroidal anti-inflammatory drugs (*n* = 3), lipid lowering agents (*n* = 8), antihypertensive drugs (*n* = 1), low dose aspirin (*n* = 1) and lifestyle modification (*n* = 1). Each point to consider differs partially or completely from recommendations previously reported for CVD risk management in RA patients from high income populations. Currently recommended CVD risk calculators do not reliably identify South African black RA patients with very high-risk atherosclerosis as represented by carotid artery plaque presence on ultrasound.

**Conclusions:**

Our findings indicate that optimal cardiovascular risk management likely differs substantially in RA patients from low or middle income compared to high income populations. There is an urgent need for future multicentre longitudinal studies on CVD risk in black African patients with RA.

## Background

Patients with rheumatoid arthritis (RA) reportedly experience a markedly increased risk of atherosclerotic cardiovascular disease (CVD) [1, 2]. Meta-analyses documented a ~ 50% increase in cardiovascular event and mortality rates in RA [3, 4]. Atherogenesis in RA remains poorly understood but clearly differs from that in non-RA persons [1, 2, 5–9]. In this regard, besides traditional cardiovascular risk factors (CVRF), disease characteristics and a genetic component are implicated in the increased atherogenesis among patients with RA [5–13]. Accordingly, CVD risk calculators based on traditional CVRF including the Framingham score and Systematic Coronary Risk Evaluation (SCORE), were found to underestimate the actual cardiovascular risk in RA [14–16]. In 2010, a European League Against Rheumatism (EULAR) task force reported recommendations for CVD risk management in RA patients [1]. A Spanish group of investigators [2] and EULAR [17] subsequently updated these recommendations in 2014 and 2016, respectively. The evidence on which these recommendations is based originates in high income populations [1, 2].

During the past ~ 40 years, the CVD incidence has decreased in high income countries [18]. By contrast, during the same period, the CVD burden has markedly increased in low and middle income countries [18]. Consequently, more than 80% of the global CVD mortality burden now occurs in low and middle income populations [19]. The increase in incident CVD in low and middle income countries is attributable to rapid urbanization and its consequent changes in epidemiologic health transition stages [19, 20]. CVRF profiles and their impact on CVD as well as cardiovascular event phenotypes differ among low or middle compared to high income populations [19–21]. In this regard, Sub-Saharan Africa comprises a region with a population of over one billion persons [22] and mostly low, to a lesser extent middle and no high income countries [23–25]. Sub-Saharan Africa is characterized not only by poverty but also by large inequalities [26]. The latter are due, at least in part, to previous colonialism and systems like apartheid [19]. Eight of the top 10 most unequal countries in the world are located in Sub-Saharan Africa [26]. Notably in the present context, although South Africa is overall a middle income country according to the World Bank [23], it is the most unequal country in the world with a Gini coefficient of 0.68 [27]. Indeed, to date, black South Africans still earn 5 times less than their white counterparts [27] and, overall, only one in four South Africans can currently be considered as either stable middle class or above in terms of means [28]. Age-standardized death rates due to CVD are currently much larger in black compared to white South Africans [29]. The estimated South African population was 57.7 million in 2018 [30]. The prevalence of RA in Sub-Saharan Africa is not dissimilar from that in high income populations [31].

Taken together, it is plausible that optimal CVD risk assessment and management differs in patients with RA from low or middle income compared to high income populations. Herein, we reviewed reported evidence on cardiovascular disease risk in South African black and white African RA patients. We subsequently aimed at formulating concise, yet comprehensive points for consideration in CVD risk management among Sub-Saharan African patients with RA, taking into consideration recent recommendations that originated in high income countries [2, 17]. The target users of this study are RA patients and health care providers that manage their disease.

## Methods

This study was performed in line with the Appraisal of Guidelines for Research instrument (AGREE II) [32]. The work group comprised (1) five rheumatologists (AS, PHD, MAG-G, SC, AES), one cardiologist (CG-J) and one epidemiologist (JL) with experience in CVD risk management in patients with RA (expert panel (EP)), (2) two patient representatives, two health professionals and one radiologist, and (3) eleven rheumatologists that regularly treat RA patients, were selected by the EP and contributed by revising the manuscript critically, providing their level of agreement for each recommendation and reading and approving the final version. The local rheumatologists (AS, PHD and AES) that formed part of the EP held several meetings throughout. Input from other EP contributors was obtained regularly through online communications.

The study was performed in 4 stages. In stage 1, AS and PHD reviewed existing American, European and Sub-Saharan African recommendations on CVD risk assessment and management in the general population and in patients with RA, and on the treatment of RA [1, 2, 17, 33–42]. In stage 2, based on the obtained information, core questions for the systematic literature review (SLR) were formulated by the EP members as guided by the Preferred Items for Systematic Reviews and Meta-analyses (PRISMA) statement [43]. In stage 3, the SLR was performed by AS and PHD up to 12/2018. The Wiley/Cochrane Library and Pubmed/Medline were searched. For our main SLR, the following search terms were used: ‘rheumatoid arthritis’, ‘Africa’ and ‘cardiovascular disease risk’ or ‘cardiovascular disease’ or ‘myocardial infarction’ or ‘stroke’ or ‘heart failure’ or ‘atherosclerosis’ or ‘endothelial dysfunction’ or ‘arterial function’ or ‘left ventricular function’, or ‘treatment’. We additionally performed a Pubmed search on meta-analyses and systematic reviews on CVD risk in the general Sub-Saharan African population using the search terms ‘Africa’, ‘cardiovascular disease’ and ‘systematic reviews’ or ‘meta-analyses’. Finally, as the SLR for the recent EULAR recommendations on cardiovascular risk management in high income populations ended in 02/2015, we searched for systematic reviews and meta-analyses on CVD risk in RA using the search terms ‘rheumatoid arthritis’, ‘cardiovascular disease’ and ‘systematic reviews’ or ‘meta-analyses’ that were published between 02/2015 and 12/2018, on Pubmed. We also identified additional relevant papers by a manual search of the reference lists of the retrieved manuscripts. Furthermore, we included 3 publications (references [22, 44, 45]) that were reported after 12/2018 in view of their perceived relevance in the present context. After removal of duplicates, titles and abstracts were screened for suitability. Full text articles were assessed for eligibility.

In stage 4, the EP formulated the points to consider. The level of evidence was determined according to the Oxford Centre of Evidence-based Medicine Levels of Evidence [46], and the strength of point to consider in agreement with 2014 Update of the EULAR standardised operating procedures for EULAR-endorsed recommendations [47]. When the underlying evidence was obtained in the general population and there was no reason to suggest that the derived points to consider would differ in Sub-Saharan African patients with RA, the level of evidence was left unaltered but the strength of point to consider was reduced by one level. The formulated points to consider were then discussed by AS, AES and PHD with the involved patient representatives, health professionals and rheumatology fellow during a separate meeting. This was done in order to facilitate their contribution to the study. Finally, the drafted manuscript was sent by email to each contributor for further intellectual input and level of agreement rating with each point to consider on a scale of 0 to 10. The obtained ratings were averaged and are presented as mean (SD). The level of evidence in support of each point to consider ranged from 1A to 3. Therefore, we did not consider it to be necessary to apply the Delphi method for reaching agreement in the current points to consider.

## Results

**Fig. 1:**
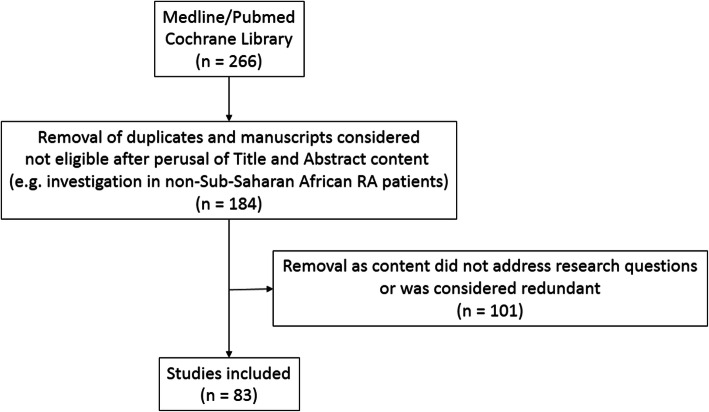
Flow chart and selection process for the main literature search. RA: rheumatoid arthritis

The core questions for our main search are presented in supplementary Table 1. The flow chart and selection process for our main SLR are given in Fig. 1. Overall, we included only manuscripts on human research and 266 articles were identified initially. Upon perusal of the Titles and Abstracts, 82 papers were excluded as they comprised of duplicates or were considered not eligible. The remaining 184 manuscripts were searched and read through. A further 101 articles were excluded as their content did not address our research questions or was considered redundant, thereby leaving us with 83 studies for inclusion in the study. All Sub-Saharan African studies that included RA patients originated in South Africa. The EP formulated 18 points to consider grouped into 6 categories that included (1) overall CVD risk assessment and management (*n* = 4) and specific interventions aimed at reducing CVD risk comprising (2) RA control with disease modifying agents anti-rheumatic drugs (DMARD), glucocorticoids and non-steroidalanti-inflammatory drugs (NSAID) (*n* = 3), (3) lipid lowering agents (*n* = 8), (4) antihypertensive agents (*n* = 1), (5) low dose aspirin (*n* = 1) and (6) lifestyle modification (*n* = 1). These are presented in Table 1.

**Table 1:** Points to consider in cardiovascular disease risk management among South African black and white patients with RA

	LoE	SoR	LoA (SD)
1. Overall CVD risk assessment and management			
1.1 We suggest to perform overall CVD risk assessment in all RA patients irrespective of population origin and socioeconomic status.	3	C	9.8 (0.5)
1.2 We suggest to perform overall CVD risk assessment upon initial presentation and subsequently at least every 5 years in RA patients at low risk, and at least yearly in those at high or very high risk.	3	C	9.7 (0.6)
1.3 We suggest that the treating rheumatologist performs primarily CVD risk assessment and management in patients with RA.	3	C	9.4 (0.9)
1.4 We suggest that (1) cardiovascular risk profiles and (2) potential benefits, (3) side effects and (4) drug-drug interactions as well as (5) patient preferences are consistently discussed between the clinician and RA patient in order to facilitate informed decision making and intervention adherence and non-discontinuation.	3	C	9.9 (0.4)
2. Specific interventions aimed at reducing CVD risk			
2.1 RA control			
2.1.1 We suggest targeting optimal RA control and, in the absence of contraindications, to consider including (hydroxy) chloroquine in DMARD regimens to reduce CVD risk in RA patients.	1B-3	A-C	9.4 (0.8)
2.1.2 We suggest considering the use of intra-articular glucocorticoids as bridge therapy upon initiating or intensifying conventional synthetic DMARD in order to reduce CVD risk in RA.	3	C	9.0 (1.4)
2.1.3 We suggest using NSAID sparingly in patients with RA, particularly amongst those at high or very high CVD risk including the presence of chronic kidney disease, and with hypertension. We recommend the preferential use of naproxen (with a proton pump inhibitor). NSAID use is not recommended in RA patients with heart failure.	1A-3	B-C	9.6 (0.7)
2.2 Lipid lowering agents			
2.2.1 In RA patients with very high CVD risk comprising (1) established CVD, (2) diabetes mellitus with target organ damage or major CVD risk factors, and (3) severe chronic kidney disease, we suggest using high dose statin therapy targeting an LDL-C level of 1.8 mmol/l. In RA patients with high CVD risk comprising (1) diabetes without target organ damage or other major CVD risk factors, (2) moderate chronic kidney disease, and (3) severe dyslipidemia (total cholesterol >/= 8.0 mmol/l or/and LDL-C level >/= 4.9 mmol/l), we suggest using moderate to high dose statin therapy targeting an LDL cholesterol level of 2.5 mmol/l.	1A-3	B-C	9.7 (0.6)
2.2.2 In RA patients without (1) established CVD, (2) diabetes, (3) moderate or severe chronic kidney disease, or (4) severe dyslipidemia, we suggest refining CVD risk assessment by carotid and femoral artery ultrasound when accessible to identify plaque presence, which represents very high CVD risk and an indication for high dose statin therapy. This is particularly important in black African RA patients.	3	C	9.5 (0.7)
2.2.3 In black RA patients without access to carotid and femoral ultrasound, we suggest to consider the presence of a CKD-EPI estimated estimated glomerular filtration rate of < 80 ml/min/1.73m^2^ as an indication for high-dose statin therapy.	3	C	9.3 (1.1)
2.2.4 In white RA patients without access to carotid and femoral ultrasound, we suggest to consider the presence of a Framingham score score of > 7.5% as an indication for high-dose statin therapy.	3	C	9.7 (0.5)
2.2.5 In black RA patients without (1) CVD, (2) diabetes, (3) moderate or severe chronic kidney disease, (4) severe dyslipidemia and (5) carotid or/and femoral plaque existence, we suggest to consider the presence of a CKD-EPI determined glomerular filtration rate of < 80 ml/min/1.73m^2^ as an indication for moderate dose statin therapy.	1A	B	9.2 (1.1)
2.2.6 In white RA patients without (1) CVD, (2) diabetes, (3) moderate or severe chronic kidney disease, (4) severe dyslipidemia and (5) carotid or/and femoral plaque, we suggest to consider the presence of a Framingham score of > 7.5% as an indication for moderate dose statin therapy.	1A	B	9.3 (0.8)
2.2.7 When affordable, we suggest the additional use of ezetimibe in RA patients when the LDL-C target is not met despite maximally tolerated statin doses. In RA patients who are intolerant to statin therapy, we suggest the alternative use of ezetimibe. When affordable, we suggest referral to a lipidologist for consideration of additional treatment with a proprotein convertase subtilisin/kexin 9 inhibitor in RA patients with established ACVD and severe dyslipidemia that have persistently high LDL-C levels despite maximally tolerated statin therapy and ezetimibe.	1B-3	B-D	9.7 (0.5)
2.2.8 We suggest lipid profile re-evaluation in RA patients subsequent to (1) obtaining low RA activity or remission due to changes in DMARD therapy, (2) major changes in life style factors and (3) one to three months after initiation or intensification of lipid lowering therapy to determine whether the LDL-C target is met. We suggest lifelong use of lipid lowering agents in RA patients with very high or high CVD risk.	1A-3	B-C	9.7 (0.6)
2.3 Antihypertensive agents			
2.3.1 We suggest considering the use of antihypertensive agents in RA patients with a SBP of 130–139 mmHg or/and DBP of 80–89 mmHg and elevated overall CVD risk, and in all patients with SBP > 140 mmHg or/and DBP > 90 mmHg. Preferred first line agents comprise angiotensin converting enzyme inhibitors, angiotensin receptor blockers, calcium channel blockers and diuretics. Preferred second line agents include spironolactone, hydralazine and minoxidil. We suggest that calcium channel blockers should be included in initial antihypertensive combination regimens in black Sub-Saharan African RA patients. We suggest yearly, monthly and three monthly blood pressure measurement in normotensive RA patients, and those with uncontrolled and controlled hypertension, respectively.	1B-3	B-C	9.6 (0.6)
2.4 Low dose aspirin use			
2.4.1 We suggest using low dose aspirin as secondary prevention in RA patients with established atherosclerotic CVD. We suggest not to use aspirin as primary intervention in RA patients without CVD.	1A-3	B-C	9.6 (0.6)
2.5 Lifestyle factors			
2.5.1 We suggest to address the importance of healthy eating habits, adequate physical activity, stress and depressive symptom control and smoking and smokeless tobacco cessation with consideration of Sub-Saharan Africa specific aggravating factors and in order to reduce CVD risk at presentation and thereafter at least yearly in patients with RA.	1A-3	B-C	9.8 (0.4)

*LoE* level of evidence, *SoR* strength of recommendation, *LoA* level of agreement; RA: rheumatoid arthritis, *CVD*: cardiovascular disease; *DMARD* disease modifying anti-rheumatic drugs., *NSAID* non-steroidal anti-inflammatory drugs *LDL-C* low-density lipoprotein cholesterol *CKD-EPI* Chronic Kidney Disease Epidemiology Collaboration

### Overall CVD risk assessment

The EULAR recommendations for cardiovascular risk management in patients with RA and other inflammatory joint diseases state that clinicians should be aware of the increased CVD risk in RA compared with the general population [17]. Since RA characteristics contribute to CVD beyond traditional CVRF in high income populations [5–8], disease specific recommendations on cardiovascular risk management are indeed justified and necessary. However, in 3 recently reported case control studies that were performed in high income countries, RA was not significantly associated with increased cardiovascular mortality among patients with a disease onset subsequent to year 2000 [48, 49] or 2003 [50]. This change over time is likely due to improved RA control. Importantly in the present context, a recent case control study in black Africans documented that the overall traditional cardiovascular risk burden, C-reactive protein concentrations and atherosclerosis burden as estimated by carotid intima-media thickness, were each similar in treated established RA compared to non-RA participants [51]. Also, C-reactive protein and interleukin-6 concentrations were not related to disease activity and severity measures in black African RA patients [51]. RA may therefore currently not impact atherosclerotic CVD in this population [51].

Sub-Saharan African black persons are reported to experience large mortality rates from cerebrovascular disease and hypertensive heart disease but a markedly low frequency of ischemic heart disease [29]. Due to their low income status, they are mostly not members of private sector medical schemes [52]. Therefore, black Africans seek medical care mostly in the public healthcare sector where resources are markedly restricted [52]. The overall traditional and non-traditional CVD risk burdens are larger in black compared to white Africans with RA [53, 54], and also larger in African RA patients that attend public compared to private healthcare facilities [55]. Further, atherosclerosis extent as represented by carotid artery intima-media thickness and the prevalence of plaque is currently as extensive in black compared to white Africans with RA [56]. We therefore suggest to perform overall CVD risk assessment in all African RA patients irrespective of population origin and socioeconomic status (point to consider (PTC) 1.1).

Similar to EULAR [17], we suggest to perform 5 yearly CVD risk evaluation in RA patients that experience low CVD risk (PTC1.2). In addition, based on findings reported in the general population [33, 34], we suggest to perfrom at least yearly CVD risk assessment in patients that are at high or very high risk (PTC1.2).

EULAR states that the rheumatologist should ensure that CVD risk management is performed in patients with RA, either by her- or himself or other healthcare providers [17]. Who should assess and manage CVD risk in African patients with RA? In the case control study among black Africans that was alluded to above [51], blood pressure values were similar and lipid concentrations were more favourable in RA patients compared to controls. Yet, antihypertensive agents and statins were prescribed in 53.9 and 40.2% (*p* = 0.02 for difference), and 19.3 and 0% of RA and control participants that sought medical care in non-rheumatology public healthcare settings, respectively [51]. We therefore suggest that, at this point in time, the rheumatologist should perform primarily CVD risk evaluation and management in African patients with RA (PTC1.3).

Compared to EULAR [17], we additionally suggest that (1) cardiovascular risk profiles and (2) potential benefits, (3) side effects and (4) drug-drug interactions as well as (5) patient preferences are discussed between the clinician and RA patient to facilitate informed decision making and intervention adherence and continuation (PTC1.4). This is in line with the 2018 Guideline on the Management of Blood Cholesterol [35]. Non-adherence to cardiovascular drug use is frequent (~ 50%) [57] and associated with increased CVD risk in the general population [58]. In this regard, Lindhardsen and colleagues reported that initiation and adherence to secondary prevention pharmacotherapy was even lower in RA compared to non-RA patients [57]. De Vera and colleagues further documented that statin discontinuation increased the risk of acute myocardial infarction in RA, as applies to the general population [59]. It is further pertinent in the present context that low income status and education level as well as depressive symptoms [60, 61], all of which are each highly prevalent in Sub-Saharan black persons [27], contribute to intervention non-adherence or/and discontinuation [62].

### Specific interventions aimed at reducing CVD risk

#### RA control

In agreement with EULAR [17], we suggest targeting optimal RA control in order to reduce CVD risk in RA (PTC2.1.1). Ample evidence was reported that cumulative inflammation is associated with CVD risk and that controlling disease activity reduces this risk in RA patients from high income populations. In Africans with RA, circulating interleukin-6 concentrations independently contribute 18% to overall endothelial activation [63]. This interleukin 6-overall endothelial activation relationship is as strong in black compared to white Africans with RA [63]. In a longitudinal study among Africans with RA, disease activity control with intra-articular methylprednisolone acetate followed by conventional synthetic DMARD therapy resulted in markedly decreased interleukin-6 levels that were strongly associated with reduced endothelial activation [64].

Since the most recent EULAR recommendations for cardiovascular disease risk management in patients with RA and other inflammatory disorders [17] were published, Rempenault and colleagues reported a systematic review and meta-analysis on the metabolic and cardiovascular benefits of hydroxychloroquine in patients with RA [65]. The use of hydroxychloroquine improved lipid profiles and reduced incident diabetes and CVD as well as insulin resistance in RA [66]. In South Africa, chloroquine can be prescribed whereas the use of hydroxychloroquine requires an additional request from the Medicine Control Council. Chloroquine is independently associated with improved lipid profiles among Africans with RA [﻿65﻿]. Given these recently reported findings, we additionally suggest to consider including (hydroxy) chloroquine in DMARD regimens to reduce CVD risk in RA patients, this in the absence of contraindications (PTC2.1.1).

Conventional synthetic DMARD take time to suppress RA activity [40]. Consequently, oral glucocorticoids are mostly recommended and used as bridge therapy upon conventional synthetic DMARD initiation or intensification [40–42]. However, in a recent meta-analysis of 34 observational studies or randomized controlled trials, glucocorticoids increased the relative risk of cardiovascular events by 47% (95% CI 34 to 60%) in RA [67]. As an alternative to oral glucocorticoids, the use of intra-articular glucocorticoids was shown to be highly effective when used as bridge therapy in several RA studies [64, 68, 69]. The use of intra-articular glucocorticoids takes advantage of its rapid non-genomic therapeutic effects [70]. In studies performed among Africans with RA, compared to oral glucocorticoids, intra-articular glucocorticoids were more favourably associated with glucose metabolism [71, 72]. As previously described, intra-articular glucocorticoid therapy in combination with conventional synthetic DMARD reduced endothelial activation in Africans with RA [64]. The use of intra-articular rather than oral glucocorticoids in RA further translates in markedly smaller cumulative doses over time [72]. In view of these reported evidences, we suggest considering the use of intra-articular glucocorticoids as bridge therapy upon initiating or intensifying conventional synthetic DMARD in order to reduce CVD risk in RA (PTC2.1.2).

In accordance with EULAR [17] and recommendations for management of cardiovascular risk in patients with RA as reported by Martin-Martinez and colleagues in 2014 [2], we suggest NSAID to be used sparingly in patients with RA, particularly in those at high or very high CVD risk including the presence of chronic kidney disease, and with hypertension (PTC2.1.3). Our literature search identified a meta-analysis of 754 trials that reported effects of NSAID on vascular and gastrointestinal events [73]. Naproxen was the only NSAID that did not increase major cardiovascular events, major coronary events, stroke and mortality [73]. All NSAID approximately doubled heart failure risk [73]. Naproxen was however most frequently associated with upper gastrointestinal complications [73]. We therefore additionally suggest to preferentially use naproxen with a proton pump inhibitor, and not to use NSAID therapy in RA patients with heart failure (PTC2.1.3).

#### Lipid lowering agents

In keeping with recommendations for CVD risk management in the general population [33–36], we suggest the use of high dose statin therapy targeting a low density lipoprotein LDL-cholesterol target of 1.8 mmol/l for RA patients in very high risk categories (PTC2.2.1). For RA patients in high CVD risk categories, the recommended LDL cholesterol target is 2.5 mmol/l. This can mostly be achieved by using moderate dose statin therapy except for in patients with severe dyslipidemia. We therefore suggest the use of moderate to high dose statin therapy for patients in high CVD risk categories (PTC2.2.1). We specifically state the different very high and high CVD risk categories in the current points to consider to encourage and facilitate their use in clinical practice.

EULAR [17] recommends the application of cardiovascular risk calculators that were produced for use in the general population, such as the Systematic Coronary Risk Evaluation (SCORE) and Framingham score. These calculators are based on major traditional CVRF profiles. Application of a 1.5 multiplication factor for the presence of RA is additionally recommended [17]. Also, screening for asymptomatic atherosclerotic plaques by use of carotid ultrasound may be considered as part of CVD risk evaluation in patients with RA [17]. In contrast to EULAR [17], we suggest to refine CVD risk assessment by carotid and femoral ultrasound when accessible, in all RA patients that do not have very high or high CVD risk profiles (PTC2.2.2). This is particularly important in black African RA patients [56, 74] (see below). We recommend including femoral in addition to carotid ultrasound as 25% of RA patients were reported to have isolated femoral artery plaque [75]. As applies to the general population [33, 34], we also suggest the use of high dose statin therapy in RA patients with carotid plaque (PTC2.2.2).

The reasons for suggesting refinement of CVD risk evaluation in the present context are as follows. Firstly, traditional CVRF including blood pressure, lipid parameters, excess adiposity and metabolic syndrome criteria are associated with atherosclerosis in white but not black Africans with RA [54, 56, 74]. Similarly, the Framingham score is associated with atherosclerosis in white but not black Africans with RA [56, 74]. Indeed, in an international 3 centre study that included RA patients without established CVD, diabetes and moderate of severe chronic kidney disease, receiver operator characteristic curve analysis revealed that the Framingham score and SCORE performed similarly in identifying white Spanish and white SSA RA patients with very high risk atherosclerosis as evidenced by carotid plaque presence, whereas the respective cardiovascular risk calculators did not discriminate black African patients with and without plaque [76]. These reported data indicate that cardiovascular risk calculators are not reliable in identifying black African RA patients with severe subclinical atherosclerotic CVD.

Secondly, cardiovascular risk calculators including the Framingham score and SCORE also often underestimate atherosclerotic CVD risk in white patients with RA [13, 14, 77, 78]. Indeed, in predominantly white Africans with RA, the mean Framingham score in those with carotid plaque is only 7% [77], which reportedly represents low CVD risk and is therefore not an indication for statin therapy. In white Spanish RA patients, 63% of those with a EULAR modified SCORE of 1 to 4% (presumed intermediate or moderate CVD risk) have carotid plaque [14]. In white Spanish women with RA and a SCORE of zero (low risk), 24% had carotid plaque [78]. These data indicate that cardiovascular risk calculators also perform sub-optimally in identifying severe atherosclerotic CVD among white RA patients. In keeping with these findings, investigations that were performed among patients from high income populations revealed that cardiovascular risk calculators perform sub-optimally in predicting incident CVD events in RA [13, 15].

Carotid and femoral artery ultrasound is an inexpensive and non-invasive investigation that is accessible in both the public and private healthcare sector in South Africa. This may however not consistently apply in other Sub-Saharan African countries. Which alternative measures are useful in estimating atherosclerotic CVD in the present context? Previous general population studies documented that black Americans are at higher risk of developing chronic kidney disease than their white counterparts [79]. Chronic kidney disease was associated with myocardial infarction and fatal coronary heart disease in black but not white Americans [80]. Even mildly reduced estimated glomerular filtration rate increases the risk of incident atherosclerotic CVD [81]. RA enhances the incidence of chronic kidney disease [82]. In a cohort study that consisted of African RA patients, the Chronic Kidney Disease Epidemiology Collaboration estimated glomerular filtration rate (CKD-EPI eGFR) was reduced to < 90 ml/min/1.73m^2^ among 49.1 and 30.6% of black and white participants, respectively [﻿82﻿]. In receiver operator characteristic curve analysis, the CKD-EPI eGFR predicted the presence of carotid artery plaque to a clinically useful extent among black RA patients [83]. The optimal cut-off value for the CKD-EPI was 82 ml/min/1.73m^2^ with a corresponding sensitivity of 42% and specificity of 91%. A CKD-EPI eGFR of < 82 ml/min/1.73m^2^ was associated with a 2.22 fold increased prevalence of carotid plaque in black African RA patients [83]. We therefore suggest that in black African patients without access to carotid ultrasound, the presence of a CKD-EPI eGFR of < 80 ml/min/1.73m^2^ is considered an indication for high dose statin therapy (PTC2.2.3).

In the 3 centre cohort study among Spanish and sub-Saharan African RA patients that was previously referred to, at a cut-off value of as low as 7.3%, the Framingham score discriminated Spanish and African white RA patients with and without carotid plaque with sensitivities and specificities of 80 and 63%, and 67 and 72%, respectively [76]. Notably, at a Framingham score cut-off value of 20% that represents high risk in the general population of high income countries, the respective sensitivities were inadequately low at 25 and 21% in Spanish and white African patients with RA, respectively [76]. We therefore suggest that in African white RA patients that do not have access to carotid ultrasound, the presence of a Framingham score of > 7.5% is considered an indication for high dose statin therapy (PTC2.2.4).

**Fig. 2:**
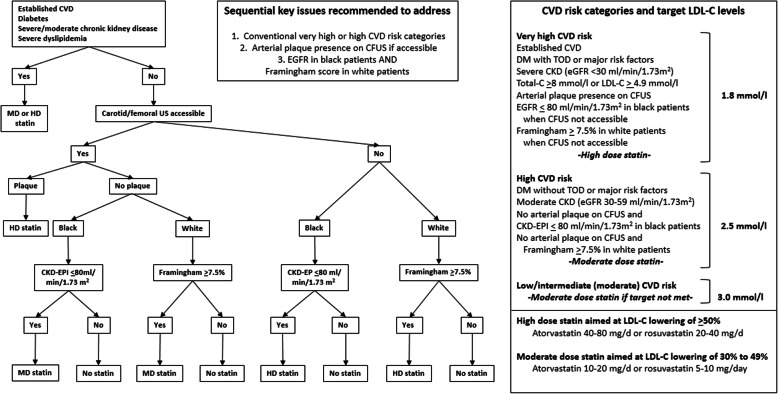
Statin therapy in Sub-Saharan African patients with RA: suggested indications, doses and targets (points to consider 2.2.1 to 2.2.6). CVD: cardiovascular disease; MD; moderate dose; HD: high dose; US: ultrasound; CKD-EPI: Chronic Kidney Disease Epidemiology Collaboration estimated glomerular filtration rate; CFUS: carotid and femoral artery ultrasound; GFR: glomerular filtration rate; ACVD: atherosclerotic cardiovascular disease; DM: diabetes mellitus; TOD: target organ damage; CKD: chronic kidney disease; C: cholesterol; LDL-C: low density lipoprotein cholesterol

Cardiovascular risk factors can cause endothelial dysfunction with consequent cardiovascular events even in the absence of marked atherosclerosis [84]. Notably in the present context, based on several meta-analyses, the recent American guidelines on the management of blood cholesterol recommend the use of moderate dose statin therapy in persons and even more so in RA patients at intermediate risk of CVD as represented by a calculated 10 year CVD risk of > 7.5 to 20% [35]. Also, a recent meta-analysis on the effects of statins on cardiovascular events in patients with mild to moderate chronic kidney disease documented that statin therapy reduced cardiovascular outcomes and total mortality as strongly in participants with mild compared to moderate chronic kidney disease [85]. In view of these evidences and given reported findings among African persons with RA [74, 83], when plaque absence is confirmed by carotid and femoral ultrasound, we suggest using moderate dose statin therapy in African black RA patients with a CKD-EPI eGFR of < 80 ml/min/1.73m^2^ (PTC2.2.5) and white patients with a Framingham score of > 7.5% (PTC2.2.6). Points to consider 2.2.1 to 2.2.6 are further illustrated in Fig. 2. Examples of moderate and high dose statin therapy are also given in Fig. 2.

Despite their proven efficacy and remarkable safety, statins are not always sufficient to reach recommended LDL-cholesterol targets in individual patients. Based on findings in the general population [35, 36], when affordable, we suggest using ezetimibe in RA patients when the LDL-C target is not reached despite maximally tolerated statin doses (PTC2.2.7). After consideration of costs involved, drug cost value and related affordability, we also suggest referral to a lipidologist for potential additional treatment with a proprotein convertase subtilisin/kexin 9 inhibitor in RA patients with established atherosclerotic CVD and severe dyslipidemia that have persistently high LDL-cholesterol levels despite maximally tolerated statin therapy and ezetimibe (R2.2.7) [35, 36]. This should be viewed in light of recently reported studies including the ODYSSEY Outcomes trial [86].

As applies to EULAR [17], we suggest lipid profile re-evaluation after obtaining low RA activity or remission due to changes in DMARD therapy (PTC2.2.8). Excess adiposity is associated with adverse lipid profiles and increased blood pressure and glucose concentrations to a similar extent in black and white African women with RA [74]. A longitudinal study among African patients with RA documented clinically relevant effects of both DMARD and dietary intervention on lipid concentrations [87]. In view of these findings together with those reported in the general population [34, 35], we additionally suggest to reassess lipid profiles subsequent to major changes in life style factors and one to 3 months after initiation or intensification of lipid lowering therapy to determine whether the LDL-cholesterol target is met (PTC2.2.8). As previously stated, lipid lowering agent discontinuation increases CVD risk in the general population [58] as well as RA patients [59]. We therefore suggest lifelong use of lipid lowering agents in RA patients with very high or high CVD risk (PTC2.2.8).

#### Antihypertensive agents

Point to consider 2.3.1 deals with the treatment of hypertension in African patients with RA. Hypertension is the main CVD risk factor in Sub-Saharan Africa [88]. A systematic analysis of data from 199 countries revealed that the age-standardized mean systolic blood pressure in 2008 was larger in Sub-Saharan Africa than in Western Europe and North-America [89]. A recent meta-analysis reported a pooled hypertension prevalence of 30% in Sub-Saharan Africa [90]. Among Sub-Saharan African persons with hypertension, overall, only 27% are aware of their hypertension status, only 18% are treated and only 7% have controlled hypertension [90]. It is therefore not unexpected that the age-standardized incidence of stroke is currently larger in Sub-Saharan Africa than in high income countries [91, 92]. Further, hypertension, rather than ischemic heart disease as applies to high income countries, is the most common cause of heart failure in Sub-Saharan Africa [93].

Hypertension is more prevalent in black compared to white and other Africans with RA [94]. Black African RA patients also experience less adequate blood pressure control than their white counterparts [83]. Blood pressure related cardiovascular and renal complications are more common and severe in black compared to age matched white patients at any blood pressure level [38].

During the recent past, controversy has arisen about hypertension definitions and target blood pressure in the general population [95]. The use of antihypertensive agents is mostly recommended for primary intervention in patients with stage 1 hypertension (systolic blood pressure (SBP) = 140 to 159 mmHg or diastolic blood pressure (DBP) = 90 to 99 mmHg) in association with elevated overall atherosclerotic CVD risk, and in all patients with a SBP > 160 or DBP > 100 mmHg. Indeed, this approach is currently recommended in South Africa [39] and is still endorsed by the 2018 European guidelines for the management of arterial hypertension [38]. By contrast, based on recently reported evidence, the 2017 American Hypertension Guideline [37] now categorizes persons with a SBP of 130 to 139 mmHg or DBP of 80 to 89 mmHg as stage 1 hypertensive patients. Accordingly, the use of antihypertensive agents for primary intervention is recommended in patients with a SBP of 130 to 139 mmHg or DBP of 80 to 89 mmHg in association with increased overall atherosclerotic CVD risk, and in all patients with a SBP > 140 mmHg or DBP > 90 mmHg [37]. Tighter blood pressure control as recommended in the 2017 American Hypertension Guideline has been questioned in (1) young patients that are at overall low CVD risk as such persons may not benefit from the respective intervention, and (2) patients with overall high CVD risk due to the presence of diabetes, chronic kidney disease, heart failure, coronary artery disease or/and advancing age. In the latter group, intensified blood pressure control may increase adverse events and CVD through the presence of impaired arterial compliance that predisposes to excessive DBP lowering. i.e. below 60 mmHg [95]. Nevertheless, considering the recent 2017 American Hypertension Guideline together with the facts that the hypertension mediated CVD risk burden is distinctly large and adequate hypertension control is currently not reached in as much as 93% of Sub-Saharan African hypertensive patients, we opted to suggest blood pressure management in RA patients as advocated in the respective recent American guidelines [37] (PCT2.3.1). In the present context, we recommend to identify elevated overall CVD risk among African patients with RA as outlined in PCT2.2.1 to 2.2.6 and Fig. 2.

In keeping with recently reported evidence on the treatment of hypertension in the general population [37–39], for primary intervention, we suggest angiotensin converting enzyme inhibitors, angiotensin receptor blockers, calcium channel blockers and diuretics as preferred first line agents, and spironolactone, hydralazine and minoxidil as preferred second line agents in African patients with RA. The use of a calcium channel blocker or diuretic in combination with an angiotensin converting enzyme inhibitor or angiotensin receptor blocker is indicated as initial therapy in most hypertensive patients except in those with low-risk grade 1 hypertension and in very old (age more than 80 years) and frail patients [38]. This should also apply particularly in patients with an autoimmune disease that is associated with increased CVD like RA. It is noteworthy that beta blockers are not included among preferred antihypertensive agents. In this regard, beta blockers can increase arterial wave reflection [96], which is associated with subclinical atherosclerosis in African patients with RA [97]. Also, angiotensin converting enzyme inhibitor or angiotensin receptor blocker use was reported to associate independently with improved glucose metabolism in Africans with RA [72].

In black patients, diuretics and calcium channel blockers are more effective than angiotensin converting enzyme inhibitors, angiotensin receptor blockers and beta blockers in preventing cardiovascular events [37]. Accordingly, the recent American hypertension guidelines recommend the inclusion of calcium channel blockers or diuretics in initial combination regimens in black hypertensive patients [37]. However, Ojji and colleagues recently reported that amlodipine with hydrochlorothiazide or perindopril was more effective than perindopril with hydrochlorothiazide at lowering blood pressure at 6 months in black Africans [44]. We therefore suggest to include a calcium channel blocker in initial antihypertensive combination regimens among black African patients with RA.

Lastly, we suggest yearly, monthly and three to six monthly blood pressure measurement in normotensive (SBP < 120 mmHg and DBP < 80 mmHg) African RA patients, and those with uncontrolled and controlled hypertension, respectively.

#### Low dose aspirin

As previously noted by Barber and colleagues [98], recommendations on the use of low dose aspirin in an attempt to reduce CVD risk among patients with RA are currently lacking. In the general population, low dose aspirin is recommended in patients with established CVD [33, 34]. The use of low dose aspirin for primary intervention of cardiovascular events, particularly in patients with high CVD risk or diabetes, has been recommended in some guidelines [45]. However, the 2016 European Guideline on CVD prevention in clinical practice [33] argues against low dose aspirin therapy for primary prevention of cardiovascular events due to the increased risk of major bleeding. In 5 randomized controlled trials that were performed subsequent to the year 2000 and formed part of a recent meta-analysis, aspirin did not reduce all-cause mortality but increased the incidence of major bleeding and intracranial haemorrhage [45]. These results were also reproduced in participants with diabetes and patients with a CVD risk > 7% [45]. A case-crossover and propensity score-matched cohort study found no protective effect of aspirin on incident myocardial infarction in RA [99]. A secondary analysis of the Prospective Randomized Evaluation of Celecoxib Vs Ibuprofen Or Naproxen (PRECISION) trial also revealed no effect of aspirin on cardiovascular event rates when used in combination with NSAID and esomeprazole, in RA [100]. Given these reported data in the general population and RA, we suggest using low dose aspirin in RA patients with but not without established atherosclerotic CVD (PTC2.4.1).

#### Lifestyle factors

Ample evidence was reported in support of the beneficial effects of body weight control, physical activity and smoking cessation in the general population [33]. Increased physical activity also improves CVRF profiles and microvascular and macrovascular function in RA [101]. In Africans with RA, dietary intervention prevented the increase in total and LDL cholesterol concentrations upon suppression of the acute phase response with conventional targeted DMARD [87]. Among African black persons, those with RA exercise less frequently but simultaneously experience less prevalent overall and abdominal obesity compared to their non-RA counterparts [51]. Nevertheless, the adiposity burden is markedly larger in black compared to other Africans with RA [94].

In a recent longitudinal multicentre study on 5638 RA patients that included African participants, smoking had the largest population attributable risk associated with incident cardiovascular events [7]. Black Africans with RA smoke infrequently [94]. However, the Gauteng Rheumatoid Evaluation and Assessment Trial (GREAT) revealed that 48% of female black participants used smokeless tobacco products in the form of inhaled snuff [102]. Blood levels of the nicotine metabolite cotinine, were as large among inhaled snuff product users as in cigarette smokers [103]. Inhaled snuff products contain heavy metals and pro-inflammatory microbial substances, particularly bacterial endotoxins that are implicated in atherogenesis [103, 104]. The use of inhaled snuff products is widespread in Sub-Saharan Africa [105].

The urbanization induced rapid epidemiological health transition in Sub-Saharan Africa is characterized by lifestyle changes that result in adverse CVD risk profiles [106]. Importantly, this process is substantially aggravated by factors that are specific to Sub-Saharan Africa and include socioeconomic and poverty related stressors, a lack of resources and primary focus on communicable diseases, low education levels, inadequate healthcare infrastructure and cultural beliefs [106].

Finally, recent meta-analyses documented that both depression and anxiety are independently associated with incident CVD event rates [107, 108]. Anxiety may increase CVD risk through reduced adherence to healthy behaviours as well as physiological mechanisms including autonomic dysfunction, inflammation, endothelial dysfunction and increased platelet aggregation [109]. The prevalence of depression and anxiety is increased in RA patients from high income countries [110, 111]. These comorbidities predict long term physical health outcomes and treatment response in RA [112]. In Sub-Saharan Africa, public healthcare attendance represents a low-income marker [113] that is associated with markedly increased tension and depressive symptom burden in patients with RA [55]. Tension scores are associated with both carotid intima-media thickness and plaque presence in black but not white Sub-Saharan Africans with RA [56].

In line with reported evidence on the role of lifestyle management aimed at reducing CVD risk in the general population and RA, we suggest to address the importance of healthy eating habits, adequate physical activity, smoking and smokeless tobacco cessation and stress and depressive symptom control with consideration of Sub-Saharan Africa specific aggravating factors and in order to reduce CVD risk at presentation and thereafter at least yearly in patients with RA (PTC2.5.1).

## Discussion

This study provides points to consider in the evaluation and management of CVD risk among black and white Sub-Saharan African patients with RA. In contrast to the EULAR recommendations for CVD risk management [17], we focused on RA and did not address cardiovascular risk in patients with ankylosing spondylitis and psoriatic arthritis. This is because the latter two diseases are distinctly uncommon in Sub-Saharan Africa [114–116] and, to our knowledge, data on CVD risk inSub-Saharan African patients with ankylosing spondylitis and psoriatic arthritis has not been reported to date.

Our evidence base included studies on CVD risk in RA patients and the general population living in Sub-Saharan Africa as well as in high income countries, the latter mostly as recently reported. Further, we perceived a need for comprehensiveness given the limited resources in the present context. Our approach resulted in the formulation of 18 points to consider, the contents of which each differ partially or completely from those of previously reported recommendations for CVD risk management in RA patients from high income populations [1, 2, 17]. The level of agreement with these points to consider among contributors was consistently high. The most striking finding is that conventional cardiovascular risk factor based algorithms, which are recommended for use in CVD risk stratification in RA and non-RA persons from high income populations [1, 2, 17], are not reliable in identifying Sub-Saharan African black RA patients with very high risk atherosclerosis [56, 76]. By contrast, the estimated glomerular filtration rate is useful to a clinically relevant extent in this context [83]. Our results support the notion that optimal cardiovascular risk management is likely to differ substantially in RA patients from low or middle income compared high income populations [19].

A most pivotal factor in reducing cardiovascular disease in RA patients is disease activity control [12, 17]. Current South African guidelines on the management of RA recommend the use of biologic agents when conventional synthetic DMARD therapy fails to adequately control disease activity [42], which is in line with the respective EULAR recommendations [40]. Financial support from medical insurances can often facilitate biological agent use in South African private healthcare patients. However, reported data indicate that despite the South African guidelines, only ~ 0.9% of RA patients that are treated in the South African public healthcare sector receive biologic DMARD [117]. This illustrates the ongoing unequal access to medical care based on socioeconomic status as one of the major problems faced by Sub-Saharan Africans. In this context also, whereas access to vascular ultrasound and the use of cardiovascular drugs, as proposed in the present set of points to consider, are currently available at no extra costs to the patient in the South African public healthcare sector, the same does not apply to many Africans living in other Sub-Saharan African countries. As indicated previously in this manuscript, under such circumstances, recently reported evidence supports the use a CKD-EPI eGFR of < 80 ml/min/1.73m^2^ and Framingham score of > 7.5% as indications for lipid lowering agent therapy when available in black and white Sub-Saharan Africans, respectively. The provision of cardiovascular drugs in patients that need it will require the involvement of governments’ ministries of health departments.

The evidence base retrieved on CVD risk in Sub-Saharan African patients with RA comprised mostly of well-designed cohort studies. However, this investigation also has important limitations and, accordingly, critical implications for future studies. Firstly, currently available studies on CVD risk in Sub-Saharan African RA patients frequently have a cross-sectional design and subclinical CVD as outcomes including atherosclerosis and endothelial dysfunction, rather than cardiovascular events. Secondly, whereas data on cardiovascular risk in black and white Sub-Saharan African patients with RA now exist, such information on those from mixed ancestry and Asian minority population groups are currently missing. Thirdly, most of the currently available evidence on CVD risk in Sub-Saharan African RA patients originates in two centres located in Johannesburg, South Africa. Given the extreme level of inequality in South Africa [27], the CVD risk burden could be expected to be similar in South African compared other Sub-Saharan black patients with RA. However, whether this is indeed the case requires further studies in which RA patients from Sub-Saharan African countries other than South Africa are included. Fourthly, there is a dearth of data arising from interventional studies. Fifthly, given the consistently high level of agreement reached in the present study, the subsequent additional application of a Delphi method for reaching further agreement may have been helpful. In this regard also, evidence obtained in general population studies from high income countries as was partially relied on in the present investigation, may not be consistently applicable in low and middle income populations. Sixthly, due to limited resources, input from non-South African EP members may have been hampered as it was obtained through regular online communications rather than in person meetings. Nevertheless, this approach proved to be workable and efficient during previous collaborative projects among members of the present EP that participated in this study (e.g. [76, 78]). These shortcomings together with the reportedly high and increasing CVD burden in low and middle income populations [18] indicate that further investigations on cardiovascular risk in Sub-Saharan African RA patients are urgently needed. We are currently initiating a multicentre multinational longitudinal study to address these issues. Further work on the potential role of biomarkers in cardiovascular risk stratification among Sub-Saharan African RA patients [19] should also be encouraged. We anticipate that a need for updating the current points to consider will arise during the coming 5 years.

## Conclusion

Taken together, the findings in the present investigation indicate that optimal cardiovascular risk management likely differs substantially in RA patients from low or middle income compared to high income populations. There is an urgent need for future multicentre longitudinal studies on CVD risk in Sub-Saharan African patients with RA. In the meantime, we hope that the present study facilitates consistent CVD risk management in Sub-Saharan African patients with RA.

## Supplementary information

**Additional file 1: Table S1.** Core questions for the main systematic literature review.

## Data Availability

Not applicable.

## Abbreviations

ACVD: Atherosclerotic cardiovascular disease
AGREE: Appraisal of Guidelines for Research and Evaluation
C: Cholesterol
CFUS: Carotid and femoral artery ultrasound
CKD-EPI: Chronic Kidney Disease Epidemiology Collaboration
CKD-EPI eGFR: Chronic Kidney Disease Epidemiology Collaboration estimated glomerular filtration rate
CVD: Cardiovascular disease
CVRF: Cardiovascular risk factors
DBD: Diastolic blood pressure
DM: Diabetes mellitus
DMARD: Disease modifying agents for rheumatic disease
EP: Expert panel
EULAR: European League Against Rheumatism
GFR: Glomerular filtration rate
GREAT: Gauteng Rheumatoid Evaluation and Assessment Trial
HD: High dose
LDL: Low density lipoprotein
LDL-C: Low density lipoprotein cholesterol
LoA: Level of agreement
LoE: Level of evidence
MD: Moderate dose
NSAID: Non-steroidal anti-inflammatory drugs
PRECISION: Prospective Randomised Evaluation of Celecoxib Vs Ibuprofen Or Naproxen
RA: Rheumatoid arthritis
SBP: Systolic blood pressure
SCORE: Systematic Coronary Risk Evaluation
SLR: Systematic literature review
SoR: Strength of recommendation
TOD: Target organ damage
US: Ultrasound
