# Supplementary material for: Points to consider in cardiovascular disease risk management among patients with rheumatoid arthritis living in South Africa, an unequal middle income country
Source: BMC Rheumatol. 2020 Jun 16;4:42. doi: 10.1186/s41927-020-00139-2 (PMC7296622; doi:10.1186/s41927-020-00139-2)
Supplement: Supplementary file 1 — Additional file 1: Table S1. Core questions for the main systematic literature review. [file 41927_2020_139_MOESM1_ESM.docx]

**Supplementary Table 1** Core questions for the main systematic literature review

__________________________________________________________________________________

1. How should CVD risk be assessed in South African patients with RA?
2. How should CVD risk be managed in South African patients with RA?
   1. What is the role of RA control with DMARD, glucocorticoids and NSAID in reducing CVD risk

among South African patients with RA?

- 1. What is the role of cardiovascular drugs including lipid lowering agents, antihypertensives and

aspirin in reducing CVD risk among South African patients with RA?

2.3 What is the role of lifestyle changes in reducing CVD risk among South African patients with RA?

__________________________________________________________________________________

CVD: cardiovascular disease; RA: rheumatoid arthritis; DMARD: disease modifying anti-rheumatic

drugs; NSAID: non-steroidal anti-inflammatory drugs.
